# Diffuse Midline Gliomas: Clinical, Diagnostic, and Therapeutic Perspectives

**DOI:** 10.3390/biomedicines14040934

**Published:** 2026-04-20

**Authors:** Sanyukta Bihari, Dia Yang, Devarshi Mukherji, Aya Haggiagi

**Affiliations:** 1 Department of Neurology, New York-Presbyterian Hospital, Columbia University Irving Medical Center, New York, NY 10032, USA; sb4579@cumc.columbia.edu (S.B.); dy2463@cumc.columbia.edu (D.Y.); 2 College of Physicians and Surgeons, Columbia University, New York, NY 10032, USA; dm4137@cumc.columbia.edu; 3 Herbert Irving Comprehensive Cancer Center, New York-Presbyterian Hospital, Columbia University Irving Medical Center, New York, NY 10032, USA

**Keywords:** diffuse midline glioma, DMG, H3 K27-altered, H3 K27M, molecular profiling, molecular markers, MRI, amino acid PET, radiomics, liquid biopsy, dordaviprone, CAR T-cell therapy, convection-enhanced delivery, clinical trials, treatment challenges

## Abstract

Diffuse midline gliomas (DMGs) are rare but highly aggressive central nervous system (CNS) tumors that can present in both pediatric and adult populations. These tumors were redefined in the 2016 WHO classification of CNS tumors based on integrated histopathological and molecular features, and were initially designated as “DMG, H3 K27M-mutant”. In the 2021 WHO update, DMGs were incorporated into the newly defined category of primarily pediatric-type diffuse high-grade gliomas, and nomenclature was changed to “DMG, H3 K27-altered” to encompass additional molecular drivers beyond the canonical H3 K27M mutation. Clinically, DMGs arise as expansile, infiltrating tumors within midline structures and may present as non-enhancing or enhancing lesions on imaging. Diagnosis is based on neuroimaging and molecular confirmation by immunohistochemistry or sequencing when tissue is available. DMGs are categorized as WHO grade 4 malignant tumors due to their aggressive biology leading to rapid and infiltrative growth. Owing to their deep and midline location, surgical resection is typically not feasible. Radiation therapy is the backbone of treatment, but there is no standard regimen of chemotherapy that has demonstrated durable efficacy. Recent progress in therapeutic approaches has led to a major breakthrough on 6 August 2025 when the U.S. Food and Drug Administration granted the accelerated approval of dordaviprone (ONC201), marking it as the first systemic therapy for progressive DMG harboring H3 K27M mutation. Other novel approaches, including chimeric antigen receptor (CAR) T-cell directed therapies and convection-enhanced delivery, are actively under investigation. We aim to comprehensively review DMGs, including the recent insights into their biology, the evolving therapeutic landscape, and the opportunities to fuel this new momentum against one of the most formidable gliomas.

## 1. Introduction

Over the past decade, significant progress in molecular profiling has reshaped our understanding of diffuse midline gliomas (DMGs). These tumors largely arise in midline structures, including the pons, thalamus, and spinal cord. DMGs were previously known as diffuse intrinsic pontine gliomas (DIPGs), a designation based on their characteristic radiographic presentation and pontine location. As stereotactic biopsy of brainstem lesions became more routine, tissue-based analyses allowed the molecular classification of these tumors and led to the identification of the H3 K27M mutation, which is present in up to 80% of DMGs [1]. In 2016, the WHO reclassified DIPGs as “DMG, H3 K27M-mutant”, recognizing the H3 K27M mutation as a key oncogenic driver [2]. The 2021 WHO classification further refined this entity to “DMG, H3 K27-altered,” broadening the definition to encompass the genetic and epigenetic mechanisms that can be disrupted in their pathologies. These tumors are defined as WHO grade 4 tumors regardless of histological features [3]. Alterations involving the H3 K27 locus have broad molecular consequences. At a high level, the mutations lead to global loss of H3 K27 trimethylation (H3 K27me3), resulting in the upregulated expression of many genes that may contribute to an oncogenic phenotype [4]. While H3 K27-altered DMGs are most commonly seen in pediatric patients, the H3 K27 alterations also occur in adult tumors and are linked to a worse prognosis compared to H3 K27 wildtype counterparts. The treatment of H3 K27-altered DMGs remains challenging. Radiation therapy (RT) has been the mainstay of treatment; however, its benefit is largely palliative, emphasizing the need for effective therapies that extend survival. This review examines the clinical and radiographic presentation of DMGs, their histological and molecular features, the recently U.S. Food and Drug Administration (FDA)-approved dordaviprone (ONC201) for recurrent disease, and ongoing therapeutic investigations including immunotherapy and targeted approaches. We also examine the key challenges that continue to limit meaningful therapeutic progress in this disease.

## 2. Methods

This narrative review was conducted through targeted searches of the PubMed database, Google Scholar, and ClinicalTrials.gov to identify the relevant literature on DMGs. Searches were performed using combinations of the terms “diffuse midline glioma,” “DMG,”, “DIPG”, “H3 K27M,” “H3 K27-altered,” “molecular profiling,” “molecular markers,” “histopathology,” “immunohistochemistry staining,” “stereotactic biopsy,” “MRI,” “amino acid PET,” “radiomics,” “liquid biopsy,” “circulating tumor DNA,” “Treatment,” “Therapy,” “radiation therapy,” “chemotherapy,” “targeted therapy,” “immunotherapy,” “convection-enhanced delivery,” “blood–brain barrier,” “tumor microenvironment,” “treatment resistance,” “therapeutic challenges,” and “clinical trials.” For the ClinicalTrials.gov search, only trials listed as active and recruiting, enrolling by invitation, or active but not recruiting were included.

Studies were included based on their relevance to the key topics addressed in this review, including diagnostic evaluation, imaging advances, molecular pathogenesis, current therapeutic strategies, investigational treatments, drug delivery approaches, and treatment resistance in H3 K27-altered DMGs. Priority was given to clinical trials, clinical cohort studies, and translational investigations with direct clinical relevance, while selected preclinical studies were included when they provided important mechanistic insights relevant to therapeutic development and resistance. Further pertinent studies were located by screening the reference sections of primary citations.

As this review aims to provide a narrative synthesis of clinical, diagnostic, and therapeutic perspectives, formal systematic review inclusion and exclusion criteria were not applied, and study selection prioritized clinical relevance and scientific impact.

## 3. Epidemiology

The 2023 Central Brain Tumor Registry of the United States documented that the yearly incidence rate of H3 K27-altered DMG in the general population is 0.6 per million persons (0.06 per 100,000) for the period 2016–2020, based on diagnostic criteria established in the 2016 WHO classification. The median age of diagnosis is 15 years (interquartile range, 7–33) [5]. A clear difference in disease prevalence exists between pediatric and adult populations. These tumors occur predominantly in children, affecting approximately 10–20 cases per million persons each year [6,7]. Roughly one in five pediatric gliomas is classified as an H3 K27-altered DMG, and these tumors account for an estimated 15% of cancer–related fatalities within the pediatric brain tumor population [8,9,10,11]. In contrast, DMGs are less common in adults, with an annual incidence of approximately 2.32 cases per million among individuals aged 20+ years [12,13,14,15]. No consistent sex predilection has been identified. Tumor location also varies by age group: pediatric DMGs most commonly arise in the brainstem, whereas adult tumors are more frequently reported in the thalamus [16,17]. Despite ongoing research, prognosis remains poor, with a median overall survival (OS) of approximately 10 months following initial diagnosis and fewer than 10% of patients reaching the two-year survival mark [18,19].

## 4. Clinical Presentation

Clinical manifestations vary according to tumor location. Headache is the most common presenting symptom, frequently progressive, and is often associated with obstructive hydrocephalus, particularly in brainstem and thalamic tumors. Associated symptoms of elevated intracranial pressure (ICP), such as nausea and emesis, are commonly reported [14]. Involvement of long tracts results in motor and sensory deficits while extension into the cerebellum can lead to dizziness, ataxia, and impaired coordination. Brainstem tumors are associated with multiple cranial neuropathies including dysarthria, dysphagia, facial weakness, and impaired extraocular movements [13]. In both children and adults with thalamic DMGs, cognitive and behavioral changes, including irritability, mood alteration, impaired attention, and neurocognitive decline, have been documented [20,21]. Although more common at progression, leptomeningeal dissemination and spread to distant regions of the neuroaxis are reported [22,23,24,25,26]. Seizures are an uncommon presenting symptom as lesions in deeper structures have less potential for cortical involvement; however, seizures can be seen as a complication of increased ICP secondary to obstructive hydrocephalus [27].

## 5. Diagnostic Evaluation

Establishing the diagnosis of DMG relies primarily on neuroimaging and tissue-based molecular analysis. Standard evaluation includes brain MRI with and without contrast, often supplemented by magnetic resonance spectroscopy (MRS), perfusion studies, and positron emission tomography (PET). MRI of the spine is obtained if there is a concern for spinal cord involvement. Lumbar puncture (LP) may be performed selectively to assess for leptomeningeal dissemination, particularly if there are suspicious symptoms and signs that are not completely explained by tumor location [28,29].

Advances in MRI and CT imaging have enabled neuronavigation techniques that reduce post-surgical complications and improve biopsy accuracy. A meta-analysis of 38 studies evaluating diagnostic yield and safety in brainstem tumor biopsies reported a diagnostic success rate of 96.2% (95% CI: 94.5–97.6%). This was accompanied by a 7.8% overall morbidity rate (95% CI: 5.6–10.2%), including instances of permanent morbidity (1.7%), and mortality (0.9%) [30]. Postprocedural complications most commonly include cranial nerve palsies hydrocephalus, intraventricular hemorrhage, and central apnea requiring prolonged intubation. In most cases, these deficits improve over weeks to months following surgery. Overall, the diagnostic yield and postoperative complication rates for brainstem tumors are comparable to those for supratentorial tumors. With ongoing refinements in neuronavigation, surgical management has expanded beyond biopsy to include partial decompression for tumors with an exophytic component [31,32,33,34,35]. As biopsies have become more routinely performed in recent years, tissue analysis has identified additional molecular markers that could serve as targeted therapies.

Immunohistochemistry (IHC) is utilized to detect H3 K27M-mutant protein using antibodies directed against the mutant epitope present in either H3.3 or H3.1 protein variants, encoded by *H3F3A* and *HIST1H3B* genes, respectively. Positive nuclear staining is highly sensitive and specific for the presence of a canonical H3 K27M mutation, especially when correlated with tumor location and histology [1,36]. IHC is also used to assess global levels of H3 K27me3, the reduction in which reflects the impaired activity of Polycomb Repressive Complex 2 (PRC2), a downstream epigenetic consequence of the H3 K27M mutation [1,37]. As per the National Comprehensive Cancer Network Guidelines^®^ (NCCN) 2026 guidelines, loss of H3 K27me3 on IHC in a midline diffuse glial tumor with “fulfillment” of appropriate histologic parameters is diagnostic of H3 K27-altered DMG [38]. However, it is important to note that IHC does not capture the full spectrum of molecular alterations encompassed by the WHO 2021 category of H3 K27-altered gliomas. Next-generation sequencing (NGS) with fusion detection or equivalent molecular profiling is recommended for the diagnosis and management of DMGs [38]. Genome-wide DNA methylation profiling may offer the potential to fill in diagnostic gaps in challenging cases although standardized reference ranges, interpretations, and the validity of techniques need to be further refined before it can be integrated as standard diagnostic work-up [39,40,41,42].

Liquid biopsy analyzing cerebrospinal fluid (CSF) or plasma offers a minimally invasive approach to molecular profiling in patients with DMG, where surgical biopsy and repeated tissue sampling are often limited. H3 K27M circulating tumor DNA (ctDNA) has been identified in the CSF and plasma of pediatric DMG patients using droplet digital polymerase chain reaction (ddPCR), with CSF yielding higher mutant allelic fractions (MAFs) compared with plasma [43]. The same group subsequently optimized liquid biopsy sensitivity and specificity across two ddPCR platforms and validated methods in different triple-matched specimen types (tumor tissue, CSF, and plasma). Following these optimization methods, they showed 100% sensitivity and specificity for H3.3 K27M detection in tumor tissue and CSF [44]. This strong concordance with tumor tissue genotyping supports the feasibility of CSF-based H3.3 K27M as a surrogate for tumor molecular diagnosis when tissue acquisition is restricted. Despite these advances in ddPCR techniques, several challenges limit the widespread implementation of liquid biopsy in clinical practice. CSF collection typically requires an LP, which in pediatric populations may necessitate general anesthesia, adding procedural complexity and risk. While plasma-based ctDNA sampling offers a less invasive alternative, it generally yields lower MAF compared to CSF, thereby reducing assay sensitivity. This difference is largely attributed to the limited transit of tumor-derived DNA into the systemic circulation across the blood–brain barrier (BBB) and the inherently low levels of ctDNA shed by primary CNS tumors [45,46]. Detection rates also vary by CSF sampling site: intraoperative collection ~100%, ventricular collection via Ommaya or shunt ~93%, and lumbar puncture ~66% [47]. Furthermore, the rarity of DMG restricts the availability of large patient cohorts necessary for the rigorous validation of these assays. This scarcity hampers the generation of reproducible datasets required for assay standardization and broader implementation, underscoring the vital importance of multicenter collaborations and expanded access to specialized biobanks.

## 6. Radiographic Features and Advanced Imaging

### 6.1. Standard Imaging

The location of these tumors is overwhelmingly within midline structures, appearing predominantly in the pons, followed by the thalamus and the spinal cord [48]. Rare cases arising in non-midline locations have been reported, primarily in adult patients, and include tumors involving supratentorial or atypical deep structures [49]. The radiographic presentation of DMGs is heterogeneous, with some tumors appearing as expansile, non-necrotic, non-enhancing lesions and others presenting as enhancing masses with variable degrees of infiltration into the surrounding parenchyma [48,50]. In the brainstem location, DMGs typically encompass more than 50% of the pontine volume and often ensheathe the basilar artery [51]. Compared with other midline high-grade gliomas (HGGs) lacking H3 K27 alterations, these tumors tend to demonstrate less prominent peritumoral vasogenic edema and more variable enhancement patterns [52].

DMGs are usually hypointense relative to normal brain parenchyma on T1-weighted imaging, although focal areas of hyperintensity can be seen in the setting of hemorrhage, the presence of cysts, or treatment-related changes [48]. On T2-weighted and fluid-attenuated inversion recovery (FLAIR) sequences, these tumors frequently appear hyperintense, reflecting diffuse infiltration and increased water content; signal heterogeneity is common and may correspond to intratumoral cysts or necrotic components [52,53]. It has also been shown that a positive T2-FLAIR mismatch sign, in which the tumor appears relatively hyperintense on T2-weighted sequence and relatively hypointense on FLAIR, can be used to differentiate H3 K27-altered DMGs from wildtype midline HGGs [54]. Diffusion weighted imaging (DWI) offers supportive data regarding tumor cellularity; DMGs are often highly cellular and thus may demonstrate restricted diffusion, but this finding is not uniform across all cases [55]. Studies have also shown that DMGs frequently demonstrate lower ADC values compared with normal brain tissue, consistent with high cellularity, though ADC values overlap with other infiltrative midline tumors, limiting specificity [50,53,56]. Additionally, the H3.3 K27M-mutant variant tends to exhibit lower ADC metrics than those for H3.1 K27M-mutant tumors [56,57].

### 6.2. Advanced Imaging

Advanced imaging modalities provide additional physiologic information that can supplement conventional MRI sequences. Perfusion-weighted imaging (PWI) enables the evaluation of the blood flow characteristics of tumors such as neovascularization, vascular permeability, and angiogenesis. Dynamic susceptibility contrast MRI (DSC-MRI), a commonly used type of PWI, assesses relative cerebral blood volume (rCBV) and relative cerebral blood flow (rCBF). These perfusion parameters have shown potential clinical relevance in the characterization of DMGs and utility in differentiating H3 K27-altered tumors from other midline HGGs [54]. In a retrospective cohort study evaluating both children and adult patients with DMGs, H3 K27M-mutant tumors demonstrated significantly higher normalized rCBV values compared with H3 K27 wildtype tumors, suggesting increased microvascular density in the mutant group. This study also showed that combining perfusion imaging with diffusion parameters improved discrimination between mutant and wildtype tumors [58]. Across histone subtypes, tumors with H3.3 K27M mutations exhibited higher rCBV values compared with those harboring an H3.1 K27M mutation [56]. Perfusion techniques, however, are not without limitations. DSC-MRI, for example, has limited efficacy for small lesions, those near large vessels, bone/air interfaces, surgical fixation devices, lesions in the spine, or near the skull base [59]. In addition to these potential structural and anatomical limitations of using DSC-MRI, at a conceptual level, brain tumors such as DMGs do not necessarily adhere to the principles upon which DSC-MRI is based. Cerebrovascular parameters measured by DSC-MRI, such as rCBV, are calculated based on the assumption that the paramagnetic contrast agent used stays within the vascular system. However, tumors tend to have abnormally permeable vasculature, which leads to the extravascular leakage of contrast agents. As a result, this phenomenon ultimately affects the validity of rCBV estimation [60]. However, post hoc leakage-correction algorithms have been created to try to eliminate this bias and are commonly used [61]. Pre-processing techniques are also necessary for DSC-MRI to address artifacts such as slice timing misalignment, geometric distortions, physiological noise, and motion [62]. Reliable techniques have been developed to reduce these distortions; nevertheless, the standardization of these correction techniques across institutions is of great necessity to enhance the diagnostic reliability of DSC-MRI [62].

MRS offers non-invasive metabolic information and is commonly used in neuro-oncology. Frequently assessed metabolites include choline (Cho), N-acetylasparatate (NAA), myo-inositol (mI), and creatine (Cr). In DMGs, an increased Cho:NAA ratio has been associated with more aggressive tumor behavior and poorer survival [63]. Conversely, increased mI:NAA ratios are associated with improved survival, potentially reflecting decreased cell turnover and lower cellular density [64]. In H3 K27-altered tumors, a decreased mI:Cr ratio was identified as a potential metabolic signature to differentiate these tumors from their wildtype counterparts [65]. Although these insights can provide utility, MRS has numerous important limitations, including restriction to specific regions of the brain, poor results for tumors near bony or air structures within the posterior fossa or spine, and poor results for necrotic or cystic lesions. MRS is typically used only for patients for whom other imaging modalities have been insufficient [59]. Additionally, MRS requires complex techniques for acquisition and subsequent post-processing, which results in long acquisition times [38]. Currently, there is a notable amount of heterogeneity in protocols across institutions, and the complexity of the post-processing steps could limit the ability for MRS to be more widely adopted [66].

Another imaging modality that capitalizes on the metabolic characteristics of the tumor is PET. ^18^F-fluorodeoxyglucose (FDG) is the most commonly used tracer, but its utility is limited in the midline because high background uptake in the normal brain tissue makes it difficult to define tumor boundaries. FDG PET also suffers from suboptimal diagnostic capability due to a lack of definitive diagnostic thresholds [66,67]. Additionally, interpretation difficulties can arise in the context of hemorrhage, radiation effects, and seizures [59].

To overcome these limitations, amino acid PET has gained significant attention as it provides a clearer “tumor-to-background” contrast which is important in DMGs given their anatomical location [68]. Several amino acid-based tracers have been studied in DMGs to varying extent, with ^18^F-fluoro-L-dihydroxyphenylalanine (F-DOPA) having the most detailed currently available survival and molecular correlation data in DMG-specific cohorts. F-DOPA PET in pediatric DMGs specifically centered in the brainstem (DIPGs) demonstrated increased uptake relative to normal brain, even in tumors with minimal or absent contrast enhancement on MRI. Quantitative analysis using the tumor-to-striatum (T/S) uptake ratio was found to be the most reliable parameter for identifying the H3 K27M mutation. In comparative analyses, a T/S ratio of >1.0 achieved an Area Under the Curve (AUC) of 0.94, outperforming advanced MRI parameters like ADC and Cho/NAA ratios in predicting the tumor. Patients with high T/S ratios also had shorter OS compared to those with lower ratios [69].

Other amino acid tracers have been evaluated to a more limited extent in smaller cohorts and mixed brainstem glioma cases. ^11^C-methionine (MET) PET was studied in a cohort of pediatric patients with DIPG and was shown to have a high diagnostic yield in distinguishing these tumors from other brainstem lesions. Regions of PET avidity at baseline also predicted sites of subsequent tumor progression on follow-up imaging, although consistent prognostic associations were not seen [70]. O-(2-[^18^F]fluoroethyl)-L-tyrosine (FET) PET has also been applied in gliomas to improve the visualization of metabolically active tumor regions beyond standard MRI sequences. In a brainstem and spinal cord glioma cohort that included two patients with DIPG, FET uptake was observed in most high-grade cases and in some instances highlighted regions of tumor activity when MRI contrast enhancement was minimal or absent [71,72]. However, although FET PET data in gliomas is growing, it is not yet FDA-approved in the United States [66]. Overall, these amino acid-based PET techniques provide an extra layer of insight in addition to FDG PET, but they also face limitations such as variability in physiological uptake of tracers, inferior spatial resolution compared to anatomical MRI, and variability in interpretation [59]. In pediatric populations specifically, amino acid tracers may have increased uptake in patients with developmental venous anomalies (DVAs). DVAs have been shown to have a higher prevalence in pediatric populations with brain tumors, which may lead to false-positive amino acid PET interpretation [73].

### 6.3. Investigational Imaging

Finally, a major area of ongoing investigation involves the use of radiomics. Radiomics is a technique that utilizes machine learning algorithms to extract large amounts of quantitative features from medical images for a variety of purposes. In the context of DMGs, multiparametric MRI-based radiomics has been developed to noninvasively predict H3 K27M mutation status, achieving an AUC value up to approximately 0.969 in some internal validation cohorts when combining features from multiple sequences and machine learning techniques [74,75]. Radiomic features derived from FLAIR and non-enhancing T-weighted imaging have shown promise in predicting progression-free survival (PFS) in a cohort of pediatric patients with DMG [76]. A primary limitation of radiomics is the significant variability in extracted features between different centers due to differences in hardware and image acquisition protocols [77]. Additionally, a lack of external or multicenter validation is a major weakness that impacts the utility of radiomics models on independent datasets [73]. Therefore, models should be built to generalize despite the variability in data, to provide real utility in clinical decision-making [78]. Efforts should be focused on implementing standardized pipelines, automating steps that are prone to operator-dependent variability, and conducting larger multicenter studies. Furthermore, the integration of technologies such as explainable artificial intelligence (XAI) algorithms could aid in standardizing the interpretation of complex data like radiomics. Traditional artificial intelligence algorithms are extremely powerful but are often a “black box” to the user. XAI algorithms provide insight into the decision-making process that led to a result. In the context of radiomics, XAI may provide explanations for which imaging features influenced the diagnostic result and describe which regions of the tumor were particularly meaningful in driving its prediction. This added feature is of great value because it allows the clinical team to verify the validity of the algorithm’s decision-making process and have better trust in the results [79].

## 7. Classification and Molecular Features

Historically, DMGs have been largely defined by the location of the tumors and radiographic appearance. Up until the early 2000s, surgical biopsy and resection were generally associated with a high risk of complications because of the deep location of these tumors. Thus, biopsy was generally not pursued, especially since it did not change management [80]. It was not until advances in stereotactic techniques lowered the risk of complications that tissue samples became more readily available to guide our understanding of these tumors [30,80].

**Figure 1:**
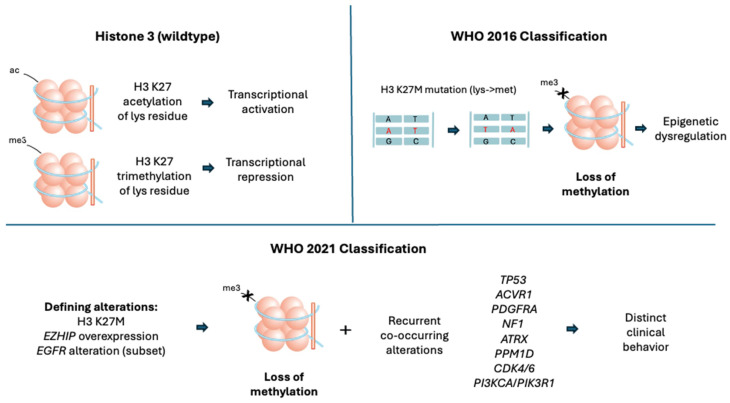
Molecular basis and WHO classification of diffuse midline glioma. The 2016 WHO classification identified the H3 K27M mutation as a defining feature of diffuse midline glioma. This lysine-to-methionine substitution inhibits Polycomb Repressive Complex 2 (PRC2) activity, resulting in a global reduction in H3 K27 trimethylation (H3 K27me3) and widespread epigenetic dysregulation. Subsequent studies demonstrated that tumors with *EZHIP* overexpression or *EGFR* alterations also result in a loss of methylation at H3, and thus the 2021 classification re-coined them from H3 K27M-mutant to H3 K27-altered to include these mutations. In addition to these defining alterations, recurrent co-occurring genetic events including *TP53*, *ACVR1*, *PDGFRA*, *NF1*, *ATRX*, *CDK4/6*, *PPM1D*, *PIK3CA*, and *PIK3R1* contribute to distinct oncogenic signaling programs and clinical behavior but are not disease-defining. Abbreviation: Lys—Lysine; Met—Methionine; *EZHIP*—Enhancer of Zeste Homologs Inhibitory Protein; *EGFR*—Epidermal Growth Factor; *TP53*—Tumor Protein 53; *ACVR1*—Activin A Receptor Type 1; *PDGFRA*—Platelet-Derived Growth Factor Receptor Alpha; *NF1*—Neurofibromin 1; *ATRX*—Alpha Thalassemia/Mental Retardation; *PPM1D*—Protein Phosphatase Mg^2+^/Mn^2+^-Dependent1D; *CDK4/6*—Cyclin-Dependent Kinase 4 and 6; *PIK3CA*—Phosphatidylinositol-4,5-Bisphosphate 3-Kinase Catalytic Subunit Alpha; *PIK3R1*—Phosphatidylinositol 3-Kinase Regulatory Subunit 1.

As we better understand the biology of DMGs, it became evident that the traditional histological classification was insufficient in clearly defining DMGs as a distinct entity. Molecular markers provided key predictive and prognostic value in determining clinical progression as well as potential therapeutic targets (Figure 1). Therefore, the 2016 WHO classification became the first to include molecular features in defining brain tumors. The 2016 WHO classification redefined DIPGs as “DMG, H3 K27M-mutant”, which incorporated H3 K27M mutation as a key defining feature. H3 K27M mutation involves the H3 histone gene that results in a substitution of lysine by methionine [2]. Approximately 80% of DMGs have this mutation in the *H3.3* (*H3F3A*) or *H3.1* (*HIST1H3B*) genes, ultimately affecting transcriptional activity [8,9,81,82].

The H3 K27M mutation affects the expression of many genes involved in tumorigenesis in complex ways that are still under investigation. In the wildtype form, the H3 lysine 27 residue is either acetylated (H3 K27ac) to increase transcriptional activity or trimethylated (H3 K27me3) by the PRC2 to repress transcription. The K27M-mutant protein acts as a dominant-negative inhibitor; it binds to and sequesters the PRC2 complex, leading to a global loss of the repressive H3 K27me3 mark across the genome [82,83,84].

While the 2016 classification appropriately identified the H3 K27M mutation as a key feature of DMGs, subsequent research identified alternative pathways that mimic this phenotype through the loss of H3 K27me3. Recent studies have demonstrated that gain-of-function mutations in the Enhancer of Zeste Homologs Inhibitory Protein (*EZHIP*) directly inhibit PRC2. The suppressed PRC2 activity leads to decreased formation of H3 K27me3 [84,85]. The resulting phenotype mimics a typical H3 K27M mutation as there is hypomethylation of H3 in both pathways. The Epidermal Growth Factor Receptor (*EGFR*) mutations, particularly exon 20 amplification, have also demonstrated an immunohistochemistry profile with H3 K27me3 loss, wildtype H3 K27M and positive *EZHIP*. Thus, *EGFR* mutation is accepted as another H3 K27-altered subtype of DMG [86,87,88]. As the 2016 WHO classification does not capture the diversity of genotypic and phenotypic mutations underlying DMGs, the updated 2021 WHO classification redefined “H3 K27M-mutant” DMGs to “H3 K27-altered” DMGs. This allows for the inclusion of additional alterations such as *EZHIP* overexpression and *EGFR* alterations in defining DMGs [3].

In addition to the defining H3 K27 alterations, the 2021 WHO classification also recognizes the frequently co-occurring molecular changes that could contribute to tumor biology and may confer a more malignant/distinct clinical phenotype. These include tumor protein 53 (*TP53*), activin A receptor type 1 (*ACVR1*), and platelet-derived growth factor receptor alpha (*PDGFRA*), which are considered clinically significant for risk stratification in H3 K27-altered DMGs. *TP53* loss is frequently associated with the *H3.3* (*H3F3A*) variant and correlates with higher-grade histology and radioresistance [22]. *ACVR1* encodes a protein involved in the regulation of bone growth and development, often co-segregates with the *H3.1* (*HIST1H3B*) variant, and has been found in a subset of DMGs, typically appearing in younger patients [89]. *PDGFRA* is a gene encoding a receptor kinase that serves as a potent oncogenic driver; its amplification is a significant prognostic marker often associated with shorter OS in DMGs [90]. Mutations in common tumor suppressor genes, other than *TP53*, including alpha thalassemia/mental retardation syndrome X-linked (*ATRX*) and neurofibromin 1 (*NF1*), are observed in a minority of DMGs. *ATRX* loss is more commonly associated with the *H3.3* (*H3F3A*) variant and frequent *TP53* co-mutation. *NF1* inactivation leads to mitogen-activated protein kinase (MAPK) pathway activation. Both are enriched in older adolescents and adult patients with thalamic and spinal cord DMGs [91].

Truncating mutations in protein phosphatase Mg^2+^/Mn^2+^-dependent 1D (*PPM1D*), or overexpression of the truncated protein (PPM1Dtr), produce a phenotype similar to *TP53* loss due to the inhibitory role of *PPM1D* in the p53 signaling pathway. The truncated form of *PPM1D* exhibits increased protein stability, leading to the dysregulation of cell cycle control and enhanced clonal proliferation in H3 K27-altered DMGs [92]. Although *PPM1D* mutations alone are insufficient to initiate oncogenesis, their occurrence alongside common H3 K27 alterations, particularly those involving the *H3.3* (*H3F3A*) variant, and *PDGFRA* can result in combined cell cycle dysregulation, suppression of apoptosis, and defective DNA damage repair, potentially leading to disease progression and more resistance to RT [92,93].

The phosphoinositide 3-kinase (PI3K) signaling pathway can be dysregulated in DMGs. Mutations within this pathway, particularly phosphatidylinositol-4,5-bisphosphate 3-kinase catalytic subunit alpha (*PIK3CA*) and phosphatidylinositol 3-kinase regulatory subunit 1 (*PIK3R1*), can enhance tumor metabolism and metastatic potential, which could contribute to increased tumor aggressiveness. Although such mutations have been reported across multiple glioma subtypes, the prevalence of their co-occurrence in H3 K27-altered DMGs is not completely clear. Clarifying this relationship is of particular interest given the availability of PI3K-targeted therapies currently used in other cancers such as breast cancer [94,95].

Major histocompatibility complex class I chain-related A (*MICA*) is a cell surface ligand that plays a critical role in T-cell activation. Specifically, in DIPG, altered *MICA* expression may have a role in evasion of T-cell recognition and activation, which may have important implications for the efficacy of CAR T-cell therapy [96].

The amplification of the myeloid cell leukemia 1 (*MCL1*) gene has also been identified as a potential therapeutic target in DMGs. *MCL1* encodes a key regulator of cell survival and apoptosis, and its overexpression promotes resistance to programmed cell death, contributing to poor clinical outcomes [97,98].

Cyclin-dependent kinase inhibitor 2A/B (*CDKN2A/B*) is a frequently detected co-alteration with *PDGFRA* amplification and is a well-established poor prognostic marker in other gliomas; however, it is rare in DMGs and its prognostic impact in H3 K27-altered tumors remains to be fully identified [91]. Similarly, cyclin-dependent kinase 4 and 6 (*CDK4/6*) amplifications are uncommon in H3K27-altered DMGs [91]. However, it is important to note that the CDK4/6 pathway dysregulation is a recurrent secondary event in DMGs and most commonly involves disruption of the CDK4/6-cyclin D-Retinoblastoma (RB) axis rather than direct activation of CDK kinases. In normal cells, cyclin D-CDK4/6 complexes phosphorylate RB1, promoting G1-S cell cycle transition, while the tumor suppressors encoded by *CDKN2A* (*p16*^INK4A^) restrain this process by inhibiting CDK4/6 activity. In some cases of DMG, this regulatory “checkpoint” is removed, leading to constitutive RB phosphorylation and uncontrolled cell cycle progression. This pathway dysregulation gained therapeutic interest in DMGs [99,100].

More recent research has also shown the significance of activating point mutations in the fibroblast growth factor receptor 1 (*FGFR1*) gene. These alterations are characterized by MAPK pathway activation and are typically seen in adult thalamic DMGs. FGFR1-mutant DMGs are notably associated with a more favorable clinical course [101,102].

Isocitrate dehydrogenase (*IDH*) mutations are not typical in H3 K27-altered tumors, and their presence should raise suspicion for a diffuse glioma; however, there are a few reported cases with simultaneous *H3.3* (*H3F3A*) and *IDH* mutations which tend to be in older patients and suggest a better prognosis [103].

Incorporating these diverse markers alongside histological and radiological features allows for a more precise definition of DMGs as a distinct clinicopathologic entity, influencing clinical practice and shaping therapeutic research targets.

## 8. Histopathology

H3 K27-altered DMGs have a wide morphological spectrum that sometimes belies their aggressive clinical nature, especially in pediatric tumors. Under the WHO 2021 classification, the diagnosis is primarily driven by the associated molecular alteration, as these tumors are assigned WHO grade 4 regardless of histological appearance. Unsurprisingly, there is a correlation between morphology and molecular markers, with H3 K27M being associated with higher-grade morphology [22]. DMGs are typically diffusely infiltrative with tumor cells interspersed among neural elements, often without a clear tumor–brain interface. Cytologically, tumor cells have astrocytic features but may occasionally display oligodendroglial-like cells. They are characterized by high cellularity, nuclear atypia, and elevated mitotic activity. They also often demonstrate perivascular pseudo-rosette patterns and high-grade microscopic features, including brisk mitosis, microvascular proliferation, and/or focal necrosis [22,104]. IHC plays a central role in diagnosis. Tumor cells show diffuse nuclear positivity for H3 K27M-mutant protein when a canonical mutation is present, along with global loss of H3 K27me3, reflecting the disruption of PRC2-mediated methylation [1,36]. Glial fibrillary acidic protein (GFAP) expression is typically retained supporting a glial lineage, while oligodendrocyte transcription factor 2 (OLIG2) expression is variable. Additional markers on IHC including ATRX loss and p53 overexpression are variably observed, reflecting underlying genetic alterations in the *ATRX* and *TP53* genes [105].

## 9. Treatment

The management of H3 K27-altered DMGs is challenging, as there is currently no globally standardized treatment regimen. Due to their deep midline location, surgical debulking and maximal resection are typically not feasible. Although molecular characterization and therapeutic development have advanced, the overall prognosis for patients with H3 K27-altered DMGs is poor. This highlights the profound unmet clinical need for more effective and durable treatments.

Symptomatic management focuses on corticosteroids or bevacizumab to mitigate peritumoral vasogenic edema, alongside CSF diversion for the treatment of obstructive hydrocephalus.

In the following sections, we review the standard of care and therapies currently under investigation for H3 K27-altered DMGs. Table 1 summarizes available data, including key efficacy signals and limitations of selected therapeutic approaches, and Table 2 outlines the numerous ongoing clinical trials investigating novel approaches.

## 10. Current Therapeutic Landscape

### 10.1. Radiation Therapy (RT)

Focal fractionated RT remains not only the established standard of care but also the only therapy consistently shown to provide clinical benefit for H3 K27-altered DMGs. Photon-based techniques such as intensity-modulated radiation therapy (IMRT) and proton beam therapy (PBT) are the most common currently used modalities. The conventional regimen involves a total dose of 54–60 gray (Gy) delivered in 1.8–2.0 Gy daily fractions over a course of approximately 6 weeks [38]. The clinical target volume (CTV) is typically defined as the T2/FLAIR abnormality plus a 1–2 cm margin to address microscopic infiltration taking into consideration the constraints of brainstem location.

Volumetric response to RT in patients with H3 K27-altered DMGs, as well as the survival benefit, has been demonstrated in several studies. A recent study reported that 7 of 11 patients achieved greater than 50% reduction in T2-FLAIR tumor volume at 3 months following IMRT [106]. Although most survival data related to RT in newly diagnosed H3 K27-altered DMGs derive from retrospective analyses, these studies consistently show that receipt of any form of RT confers a significant survival benefit compared with patients who did not receive RT or who received chemotherapy alone [13,107]. Despite these responses, RT is not a durable treatment for the aggressive and heterogenous pathology of H3 K27-altered DMGs. In the same study, nine out of 11 patients eventually experienced disease relapse, frequently with leptomeningeal and subependymal dissemination [106]. Moreover, the intrinsic genetic and epigenetic aberrancies characteristic of DMGs contribute to the dysregulation of cell cycle checkpoints and DNA damage repair pathways that are essential for regulating the damage produced by RT, thereby promoting radioresistance [108]. This biology of DMGs is reflected clinically by the relatively short PFS of approximately 7–10 months following RT [13].

While the biological impact of RT is generally consistent across age groups, the delivery techniques and long-term considerations vary between pediatric and adult patients. Comparative data between these RT modalities are nuanced; a large-scale retrospective analysis compared the outcomes between patients with gliomas treated with PBT versus IMRT and suggested that patients treated with PBT had improved median and 5-year OS [109]. Conversely, a retrospective study from Japan involving pediatric patients with newly diagnosed DIPG found that PBT did not yield superior survival outcomes compared to a historical cohort [110]. Despite these survival data, the choice of modality is often influenced by factors including patient age, tumor proximity to critical structures, and the availability of a proton center. PBT offers a distinct dosimetric advantage by eliminating the “exit dose” associated with photons, thereby sparing radiation to healthy surrounding brain tissue [38]. This is particularly important in pediatric patients, who face higher risk of long-term cognitive impairment, endocrine dysfunction, and risk of secondary malignancies [111].

Alternative fractionation schedules have also been explored to reduce treatment burden while maintaining therapeutic benefit. In a randomized noninferiority trial including 253 pediatric patients with DIPG, two hypofractionated regimens (39 Gy in 13 fractions and 45 Gy in 15 fractions) were compared with conventional fractionation (54 Gy in 30 fractions). Median OS was similar across treatment groups (9.8 months, 8.2 months, and 8.7 months, respectively). The acute and delayed toxicity rates were also comparable between treatment arms, suggesting that shorter treatment courses may reduce treatment burden in selected patients [112].

Re-irradiation (reRT) at time of progression can be considered as a potential palliative option. Several retrospective studies and a Phase 2 trial have demonstrated that focal reRT can provide temporary neurologic improvement and modest survival extension in selected patients with progressive DMG; however, the duration of benefit is typically limited and must be weighed against risks of cumulative radiation toxicity [113,114].

### 10.2. Chemotherapy (Limited)

Alkylating agents, such as temozolomide and lomustine, can be used concurrently with RT or in the adjuvant setting in both adult and pediatric populations. The NCCN guidelines for adult HGGs state that for methylguanine-DNA methyltransferase (*MGMT*) promoter unmethylated H3-mutated HGGs, treatment can include concurrent and adjuvant temozolomide, although this is a category 2B recommendation [59]. In the pediatric population, the use of temozolomide or lomustine concurrently with RT or in the adjuvant setting is a category 2A recommendation for non-pontine tumors, whereas enrollment in a clinical trial or RT alone is recommended for pontine locations (DIPGs) [38]. However, the evidence supporting the use of alkylating agents in adult and pediatric patients with non-pontine DMGs is derived largely from retrospective studies and case series rather than prospective randomized trials [115,116]. In children with pontine DMG, the addition of temozolomide to RT has not demonstrated survival benefit in clinical trials when compared to historical controls and in an open-label randomized controlled trial of hypofractionated RT with or without temozolomide [117,118]. Because these tumors are nearly universally unmethylated at the *MGMT* promoter [119] and are well protected by the BBB, they lack the chemosensitivity to traditional alkylating agents seen in other HGGs.

Historically, chemotherapy regimens using platinum-based agents or topoisomeriase inhibitors have been used to treat pediatric HGG, including DIPGs. However, many studies evaluating their efficacy in pediatric population were conducted before routine testing for the H3 K27M mutation became standard practice. Consequently, translating survival outcomes from these earlier studies into the current molecularly defined era of pediatric HGGs is challenging. Overall, conventional cytotoxic chemotherapy has not demonstrated consistent survival benefit in patients with DMG.

### 10.3. Dordaviprone

In August 2025, the FDA granted accelerated approval to dordaviprone (ONC201, Modeyso^TM^) as the first and only systemic treatment for progressive DMGs harboring an H3 K27M mutation. Following the approval, dordaviprone was integrated as a category 2A recommendation in the NCCN guidelines [38]. Dordaviprone is a first-in-class, CNS-penetrant oral impiridone that was initially patented in the 1970s as a tumor necrosis factor (TNF)-related apoptosis-inducing ligand (TRAIL). However, its specific efficacy in H3 K27M-mutant cells is primarily driven by more upstream mechanisms, acting as a selective, high-affinity antagonist of the dopamine receptor D2 (DRD2) and by simultaneously functioning as an allosteric agonist of the mitochondrial protease caseinolytic protease P (ClpP). DRD2 is a G-protein-coupled receptor that is often overexpressed in cancers, including glioblastoma (GBM) and DMGs [120,121]. This antagonism has been shown to inactivate the “pro-survival” protein kinase B (Akt) and MAPK signaling pathway [121]. ClpP hyperactivation disrupts mitochondrial oxidative phosphorylation and triggers an integrated stress response (ISR), leading to tumor cell death [122].

The identification of responders to dordaviprone in a clinical trial designed for molecularly unselected GBM showed a unique sensitivity signal in H3 K27M-mutant tumors [123]. This signal was confirmed in subsequent data [122,124,125]. More recently, in 2024, an integrated pooled analysis from five different studies (four clinical trials and one expanded access protocol) was published [126]. While this pooled approach allowed the evaluation of a larger cohort in a rare disease, it also introduces potential heterogeneity in patient selection and treatment context given the inclusion of multiple study designs and an expanded access program. A total of 374 patients were evaluated, of which 50 patients met the prespecified inclusion criteria. It is important to note that patients with pontine and spinal tumors as well as leptomeningeal dissemination were ineligible due to the difficulty of measuring response in these cases. These exclusions resulted in a more selected population and may limit the generalizability of the findings to the broader DMG patient population, particularly given that pontine tumors represent a substantial proportion of cases. Patients were also excluded if H3 27KM status was negative or unknown, if there was absence of progressive or measurable disease, or if they were treated with RT less than 90 days from enrollment. All patients had progressed through RT and most had received prior chemotherapy. Most patients except four were adults between the ages of 18 and 40 and had a Karnofsky/Lansky Performance Status (KPS/LPS) of at least 60. The predominance of adult patients is notable, as DMG more commonly affects pediatric populations, which may further influence the applicability of these findings across age groups.

Using the Response Assessment in Neuro-Oncology-High-Grade Glioma (RANO-HGG) criteria, the primary end point of overall response rate (ORR) was 20%, including one complete response and nine partial responses. Disease control rate (DCR) was 40%. The median duration of response (DOR) was 11.2 months. PFS at 6 months was 35.1% and median OS was 13.7 months [126]. The corticosteroid response rate was 46.7% and KPS/LPS response was 20.6%. Treatment-related adverse events (TRAEs) occurred in 60% of patients, with fatigue (34%), nausea (18%), and lymphopenia (14%) being the most common side effects. TRAE Grade 3 toxicity occurred in 20% of the participants. There was no Grade 4 toxicity or deaths. Dose reduction/treatment interruption occurred in one patient (2%). Although these findings demonstrate clinical activity and a favorable tolerability profile, the ORR of 20% indicates that the majority of patients do not achieve an objective radiographic response. The interpretation of imaging response in DMG is also complex, as post-RT treatment-related changes may confound radiographic assessment. Furthermore, these data were derived from non-randomized studies, and confirmation of clinical benefit will ultimately depend on prospective randomized trials such as the ongoing ACTION trial (Table 1), an international placebo-controlled Phase 3 study evaluating OS and PFS in pediatric and adult patients with newly diagnosed H3 K27-altered DMG immediately following completion of initial RT [127].

## 11. Investigational Therapies

### 11.1. Imipridones

ONC206 is a second-generation imipridone designed to enhance the therapeutic ceiling of this drug class. While it shares the DRD2 antagonism and ClpP agonism of its predecessor, ONC206 features a chemical modification, a difluorobenzyl substituent, that results in a tenfold higher binding affinity for the ClpP protease. This increased potency leads to a stronger ISR in H3 K27M-mutant cells and has been shown in preclinical settings to reduce the proliferation and viability of patient-derived DMG primary cells in vitro [114]. There are currently two actively accruing Phase 1 clinical trials evaluating the effects and best dose of ONC206 in patients with newly diagnosed or recurrent DMGs as a monotherapy or in combination with RT (Table 1).

ONC212 is another second-generation imipridone which, in comparison to dordaviprone and ONC206, has a distinct pharmacological target as a potent GPR132 receptor agonist in addition to its shared ClpP-mediated disruption. It has been studied in multiple cancer types, including pancreatic cancer, melanoma, and hematological malignancies, and has shown anti-cancer effects [115,116]. Though it has yet to be formally evaluated in central nervous system tumors, given its high potency and the shared metabolic dependencies of H3 K27-altered cells, it warrants further investigation in the context of DMG.

### 11.2. Immunotherapy

The unique tumor microenvironment of H3 K27-altered DMGs represents a critical therapeutic target. Characterized by a relative paucity of tumor infiltrating lymphocytes and proinflammatory molecules, this “immune cold” state has historically limited the efficacy of traditional immunotherapies [117]. A single-arm meta-analysis of anti-programmed cell death-1 (PD-1) and anti-programmed cell death ligand 1 (PD-L1) therapies of 2321 patients with gliomas, including DMGs, found that immune checkpoint inhibitors did not improve OS or PFS. Moreover, the pooled objective response rate was only 10% [128]. Another single-institution retrospective study that compared PFS and OS of children with recurrent DIPG who received reRT alone or in combination with PD-1 inhibition using nivolumab reported a nonsignificant improvement in OS with the combination regimen [129]. To circumvent this barrier, studies evaluated the use of chimeric antigen receptor (CAR) T-cell therapy to bypass endogenous immune suppression. CAR T-cells are engineered with a specific antigen recognition domain that, upon binding to tumor cell surface, triggers intracellular cascade leading to T-cell engagement, the release of pro-inflammatory cytokines, and subsequently tumor destruction. An important study published in 2024 evaluated CAR T-cells targeting the disialoganglioside 2 (GD2) antigen, which is intensely expressed on these tumors [130]. Among 11 patients (aged 4–30 years) with biopsy-confirmed H3 K27M-mutated tumors in the brainstem or spinal cord, 9 patients experienced clinical benefit, radiographic benefit, or both following intravenous (IV) and subsequent intracerebroventricular (ICV) administration. One patient achieved a sustained complete response exceeding 30 months after study enrollment. Cytokine release syndrome (CRS) occurred in all 11 patients exclusively after IV administration of CAR T-cells, and a greater incidence of dose-limiting toxicity occurred at the higher IV dose level. Among the patients who received subsequent ICV CAR T-cells, no dose-limiting toxicities occurred. Though no immune effector cell-associated neurotoxicity syndrome (ICANS) was observed after ICV infusions, all patients experienced transient tumor inflammation-associated neurotoxicity (TIAN) which is distinct from ICANS as ICANS is related to a systemic immune activation, whereas TIAN is localized peritumoral inflammation that leads to neurological symptoms specific to the tumor’s anatomical location. While the study’s cohort was small, excluded thalamic involvement, and was limited by adverse events, it provided a robust proof-of-concept for tumor reduction via cellular engineering.

Separately, there is an actively enrolling novel Phase 1 study that involves the world’s first quad-targeting CAR-T cell. These cells are engineered to express receptors that simultaneously bind to B7-H3 (CD276), EGFR806, human epidermal growth factor receptor 2 (HER2) and interleukin-13 receptor alpha 2 (IL-13 zetakine), and are delivered via direct intraventricular delivery to maximize local exposure while minimizing systemic toxicity [119].

In addition to cellular therapies, the utility of peptide vaccines has been investigated to prime the immune system against the H3 K27M neoantigen. One study included eight adult patients with progressive disease who had previously been treated with RT and chemotherapy [120]. Five out of eight patients were administered long H3 K27M peptide vaccine concurrently with antiPD-1 treatment, and one out of eight patients received concurrent alkylating chemotherapy; out of the two patients who received solely the vaccine, one patient achieved a sustained complete remission for over 2.5 years. Although these individual responses are compelling, larger prospective cohorts evaluating the vaccine, ideally as monotherapy, are necessary to delineate the specific survival benefit of peptide-based therapeutic approaches in H3 K27-altered tumors.

### 11.3. Targeted Therapies

#### 11.3.1. Epigenetic Therapies

Histone deacetylase (HDAC) inhibitors are considered a high-priority therapeutic class in DMGs due to their potential ability to counteract the global epigenetic disruption characteristic of H3 K27-altered tumors. Loss of H3 K27me3 leads to a compensatory increase in H3 K27 acetylation which “hijacks” the transcriptional landscape toward an oncogenic state [82,83,84]. HDAC inhibitors such as panobinostat function by blocking the enzymes responsible for removing acetyl groups, thereby attempting to “reset” these abnormal expression patterns [131]. Panobinostat has demonstrated preclinical efficacy by showing growth inhibition and survival benefit in patient-derived xenograft and genetically engineered models [132,133]. The data on its ability to penetrate the BBB is mixed. One study showed that panobinostat achieved therapeutic levels in the brainstem of a murine model [134], while a separate study in a non-human primate model reported low CSF penetration, indicating a limited BBB permeability in higher-order species [135]. These contrasting results may reflect species differences in BBB physiology and drug efflux transporter activity. To overcome the potential of poor CNS penetration and mitigate the high risk of systemic side effects that limit its full therapeutic potential, the convection-enhanced delivery (CED) of MTX110, a water-soluble formulation of panobinostat, has been investigated. By delivering the agent directly into the tumor interstitial space, this method has demonstrated a manageable safety profile in two Phase 1 clinical trials and a median OS of 26.1 months, representing a substantial improvement over historical benchmarks [136,137]. Additionally, second-generation HDAC inhibitors have been shown to have superior pharmacokinetic profiles; they include quisinostat, which is a potent hydroxamic acid-based inhibitor that is highly brain-penetrant and has been shown to induce significant histone hyperacetylation in DMG models [138,139]. Similarly, romidepsin showed high potency and provided prolonged tumor control in vivo [138].

#### 11.3.2. Cell Cycle Pathway Inhibition

The dysregulation of the CDK4/6 pathway, which most commonly involves the disruption of the CDK4/6-cyclin D-RB axis, is a recurrent secondary event in H3 K27-altered DMGs [91] and has thus been a therapeutic target. However, despite the robust biological rationale, clinical success with first-generation inhibitors like ribociclib has been modest. A Phase 1/2 study of ribociclib in pediatric DMGs was limited by its small sample size and poor clinical response, with some patients even exhibiting increased necrotic tumor volume as a treatment effect [140]. Limited CNS penetration and the rapid development of resistance could explain this result. Abemaciclib, which has excellent CNS penetration, is currently being evaluated in an active Phase 1 clinical trial for children and young adults with DMG. This study utilizes continuous microdialysis sampling for up to 48 h post-surgery to provide definitive human data on target engagement and drug concentration within the midline interstitial space (Table 1). Synergistic combinations including CDK4/6 inhibitors with dordaviprone are also being evaluated in an ongoing clinical trial (Table 1).

#### 11.3.3. FGFR Pathway Inhibition

As discussed previously, *FGFR1*-activating point mutations are particularly prevalent in adult thalamic tumors and lead to activation of the MAPK pathway, making them attractive targets for precision-based inhibition. Infigratinib, an oral FGFR1-3 inhibitor, has shown efficacy in a Phase 2 clinical trial that included 26 adult patients with *FGFR*-altered recurrent gliomas [141]. Out of all the enrolled individuals, four had disease control lasting longer than a year. Three of these responders had tumors harboring activating *FGFR1* point mutations or *FGFR3* point mutations; one had an *FGFR3*-tranforming acidic coiled-coil protein 3 (*TACC3*) fusion. The FIGHT-207 trial, a Phase 2 study, evaluated another oral FGFR1-3 inhibitor, pemigatinib, in patients with advanced solid tumor malignancies. The study enrolled 107 patients, 13 of which had unresectable CNS tumors including GBM. Within this CNS cohort, five patients demonstrated a reduction from baseline in target lesion size, three patients had objective response per RANO-HGG criteria, and three patients had stable disease [142].

#### 11.3.4. PDGFRA Inhibition

Alterations in *PDGFRA*, including activating mutations and amplifications, occur in a subset of patients particularly in pediatric cases. These alterations contribute to tumor proliferation and survival through the activation of downstream PI3K and MAPK pathways [143,144]. Targeting PDGFRA with selective inhibitors, such as dasatinib, has been explored in preclinical DMG models, demonstrating reduced proliferation and partial tumor regression, but this did not translate into clear clinical benefit [145,146]. Recent preclinical work and a small clinical trial (n = 8) showed that the highly selective CNS-penetrable PDGFRA inhibitor avapritinib exhibited activity against PDGFRA-altered glioma models and three patients on the study had disease response [147]. Larger studies on avapritinib are needed to confirm this signal of clinical activity and to further evaluate its safety profile.

#### 11.3.5. EZH2 Inhibition

Preclinical data regarding tazemetostat, an oral selective EZH2 inhibitor that was FDA-approved for epithelioid sarcoma in 2020, remain conflicting. In a study published in 2017, small-molecule EZH2 inhibitors abolished tumor cell growth in mouse models of DIPG with H3 K27M mutations by disrupting the residual activity of the PRC2 complex [148]. However, subsequent research from 2022 suggests that EZH2 may paradoxically function as a tumor suppressor in certain DMG contexts where further loss of its catalytic activity accelerates epigenetic instability [149], in which case the efficacy of tazemetostat may not translate clinically.

#### 11.3.6. Nuclear Export Inhibition

Selinexor is a highly CNS-penetrant selective inhibitor of exportin-1 (XPO1) and has shown antitumor activity in recurrent GBM [150]. While currently FDA-approved for the treatment of refractory multiple myeloma and diffuse large B-cell lymphoma, its efficacy in pediatric neuro-oncology was recently investigated in a Phase 1 trial for recurrent/refractory solid and CNS tumors [151]. Though there was only one subject enrolled in the study with H3 K27-altered DMG, that subject was among the eight who had stable disease after nine cycles of selinexor. Selinexor is currently being investigated in combination with RT in patients with DMGs (Table 1).

#### 11.3.7. Other Investigated Targets

Pathway inhibitors targeting vascular endothelial growth factor receptor 2 (*VEGFR2*) and *EGFR* were investigated as potential therapies in children with DMGs, specifically those centered in the brainstem, but did not show a significant improvement in clinical outcomes despite prior RT [152].

### 11.4. Enhanced CNS Delivery Methods

#### 11.4.1. Convection-Enhanced Delivery (CED)

The physiological barriers protecting the midline, particularly the BBB and the high interstitial fluid pressure within the tumor, can render systemic therapies ineffective [153,154,155]. To overcome these barriers, the integration of CED with novel drugs has gained interest in DMGs. CED utilizes a continuous low-pressure gradient to drive therapeutics directly into the tumor interstitial space. It relies on bulk flow (convection) rather than simple diffusion, which is often limited by a drug’s molecular weight and concentration gradient [156]. This pressure driven mechanism allows for the distribution of drugs (including high-molecular weight agents) across relatively large volumes, achieving therapeutic concentrations at the site of disease while minimizing systemic exposure [156,157]. An important Phase 1 trial in 2018 evaluated the feasibility and safety of CED in the brainstem. This study enrolled patients aged 3–21 years with DMGs centered in the brainstem (DIPGs) who previously received RT. A radiolabeled antibody, [^124^I]-8H9, which binds the surface antigen B7-H3 that is highly expressed in DIPGs, was delivered to the central region of the tumor via a stereotactically placed infusion catheter. The study demonstrated high local drug concentrations with no dose-limiting toxicities (DLTs) [158]. The same group more recently published results of a different Phase 1 trial evaluating the first-in-human theranostatic use of a [^124^I]- radiopharmaceutical (^124^I-omburtamab) both as treatment and imaging guidance tracer to track drug distribution in real time via PET scans. The trial identified the maximum tolerated activity level [159]. Chronic CED with intermittent long-term delivery to maintain therapeutic drug concentrations is another area of active investigation given the critical limitation of a single-session infusions as the treatment agents get cleared rapidly from the interstitial space. Using an implantable access port connected to an indwelling catheter, chronic CED is feasible as shown by the recently published clinical trials using the aforementioned MTX110 (aqueous panobinostat) [136,137].

#### 11.4.2. Focused Ultrasound (FUS)

FUS is a non-invasive method with the potential to address the challenges posted by the BBB in DMGs. By combining low-frequency acoustic radiation with intravenously administered microbubbles, FUS induces rhythmic contraction of microbubbles, allowing for the transient local disruption of tight junctions and the opening of the BBB. This localized opening facilitates the delivery of systemic drugs into the brainstem and thalamus where otherwise it might not be feasible due to their molecular weight or polarity. A recent prospective single-arm feasibility clinical trial involved pediatric patients with clinical or radiological progression of DMG who had previously been treated with RT and anti-cancer systemic therapies. The patients underwent repeated neuronavigation guided-FUS treatments, and BBB opening was confirmed with contrast MRI. Subsequently, patients were dosed with panobinostat. Overall, FUS treatment was well tolerated without the need for sedation or invasive surgery, and all enrolled patients achieved confirmed BBB opening [160]. There is an ongoing clinical trial evaluating the synergy of FUS with oral etoposide to maximize the therapeutic efficacy (Table 1).

#### 11.4.3. Intraventricular Delivery

ICV administration via an Ommaya reservoir has been increasingly adopted in CAR T-cell therapy trials to bypass the vascular barriers and achieve more uniform distribution across the leptomeningeal spaces and midline structures while reducing the systemic cytokine burden [161].

#### 11.4.4. Intranasal Delivery

Intranasal drug delivery is being evaluated in DMGs to bypass the BBB by utilizing the olfactory and trigeminal nerve pathways. These cranial nerves provide a direct anatomical conduit from the nasal epithelium to the brain brainstem, potentially allowing therapeutics to avoid systemic circulation and first-pass metabolism. This method is primarily in the very early phases of investigation in DMGs [162,163].

## 12. Challenges and Limitations in Therapeutic Development

Despite increasing therapeutic investigation, meaningful clinical progress in H3 K27-altered DMGs remains limited by multiple interconnected challenges spanning translational biology, drug delivery, preclinical modeling, and clinical trial design. These barriers contribute to the high rate of treatment failure observed in clinical trials, even when preclinical data are encouraging.

### 12.1. Drug Delivery and Translational Barriers

The failure of preclinical efficacy to translate into meaningful in vivo activity is a central challenge in DMG therapeutic development. This disconnect reflects, in part, inadequate intratumoral drug exposure as well as the limited ability of preclinical systems to reliably predict therapeutic response in patients.

Suboptimal penetration across the BBB is a key contributor to this gap. Brain penetration of EZH2 inhibitors has been shown to be severely limited in mouse models [164]. Similarly, a preclinical study evaluating the efficacy of the HDAC inhibitor panobinostat demonstrated that, although drug concentrations were higher in the genetically engineered mouse model (GEMM) of a brainstem glioma driven by H3 K27M expression compared with normal cerebral cortical tissue, this increase was likely due to compromised BBB in the GEMM [133], rather than effective drug delivery across an intact BBB, as would be expected in patients with DMGs.

CED offers a promising solution to circumvent the challenge of BBB penetration. In support of this approach, a study evaluated the delivery of an EZH2 inhibitor in human DIPG xenograft models compared to intraperitoneal and CED administration. The study demonstrated that, following systemic administration, the concentration of the EZH2 inhibitor in the brainstem tumor was only 3.74% of serum concentration, indicating that only a minimal amount of the drug penetrated through the BBB [165]. In contrast, CED resulted in tumor growth inhibition and significantly prolonged the survival of DIPG xenograft-bearing mice compared with those receiving the EZH2 inhibitor systemically [165].

Translational challenges extend beyond drug delivery. For both HDAC and EZH2 inhibitors, context-dependent biological effects may limit clinical efficacy despite promising preclinical activity. HDAC inhibitors have been shown to paradoxically activate transcriptional programs that enhance tumor adaptability and promote invasiveness [166]. Moreover, as discussed earlier, EZH2 may have a tumor suppressor function, and its inhibition may lead to paradoxical tumorigenesis [149].

Combination drug therapies may represent a strategy to overcome these limitations. Several preclinical studies using HDAC inhibitors in combination with proteasome inhibitors, transcription factors inhibitors, and impiridone-based combinations (e.g., dordaviprone plus tazemetostat) have shown promising results, including tumor growth reduction, metabolic catastrophe, and activation of an integrated stress response [98]. Clinical trials evaluating combination approaches are ongoing, such as NCT04943848 and NCT05009992 (Table 2).

### 12.2. Limitations of Preclinical Models

The preclinical models themselves cannot accurately reproduce the heterogeneity of DMGs in regard to their anatomical location, epigenetic alterations, and tumor microenvironment [82]. Traditional in vitro systems, including established cell lines and two-dimensional cultures, often fail to capture the complex spatial organization and tumor-microenvironment interactions present in vivo. However, efforts are being made to further refine preclinical models to more appropriately represent DMGs in humans. Patient-derived xenograft (PDX) and GEMMs have been developed to better recapitulate key molecular alterations such as H3 K27M mutation and co-occurring genetic alterations, but these models still incompletely reproduce the full spectrum of tumor heterogeneity observed in patients [167,168]. Additional models, for example, brain slices derived from rodents or transgenic mice, can more accurately preserve the cytoarchitecture of the tumor tissue into which glioma stem cells can be introduced [152]. Brain organoids, which are patient-derived or engineered three-dimensional brain tumor models, can also better replicate the in vivo environment by maintaining the inherent genetic and molecular heterogeneity of DMGs [152]. These advanced models provide opportunities to study tumor biology and therapeutic response interactions in systems that more closely approximate human disease.

### 12.3. Clinical Trial Design Challenges

Clinical trial design is another major limitation in advancing therapeutic development. Before the molecularly defined era, clinical trials unknowingly enrolled patients with the now recognized H3 K27-altered DMGs within the heterogeneous “HGG” cohort. This likely led to the investigation of therapies that were not efficacious for the distinct molecular biology and genetic drivers of tumors that are now recognized as biologically separate entities. In the current molecularly defined era, the rarity of DMG and the typically short OS associated with this disease make it challenging to conduct adequately powered randomized controlled trials. Moreover, there is a predominance of pediatric data in DMG research largely due to a higher disease incidence in the pediatric cohort and the historically poorer prognosis compared with adult patients, necessitating an urgency for new therapeutics in the pediatric population. Studies have also shown that the tumor biology of adults with DMGs is distinct from the pediatric population [169], and this difference limits the direct translation and generalizability of pediatric data to adult DMGs.

Age-specific therapeutic strategies, as well as multi-institutional clinical trial networks, can address enrollment challenges and facilitate therapeutic development. Innovative clinical trial designs may also help overcome the limitations imposed by small patient populations. Adaptive trial designs allow for preplanned modifications such as arm expansion, early termination for futility, or dose adjustments based on interim analyses, thereby improving trial efficiency and reducing the number of patients required to reach meaningful conclusions. Similarly, basket trials that enroll patients based on shared molecular alterations rather than tumor location may provide an opportunity to investigate targeted therapies across multiple tumor types, including H3 K27-altered DMGs. Platform trials that simultaneously evaluate multiple therapeutic agents within a unified trial infrastructure can also further accelerate drug development by allowing ineffective treatments to be rapidly discontinued while promising therapies are expanded or added for further evaluation. Platform trials frequently incorporate adaptive design features too. Such trials are currently being conducted in DMG, for example, NCT05009992 (Table 2). Given the rarity of DMGs, continued progress in therapeutic development will likely depend on global collaboration and coordinated international efforts capable of pooling patient populations and harmonizing trial protocols across institutions.

### 12.4. Tumor-Intrinsic Resistance Mechanisms

Cancer therapy resistance can be broadly categorized into intrinsic and acquired resistance. Intrinsic resistance refers to pre-existing tumor features that limit therapeutic efficacy, whereas acquired resistance emerges due to evolutionary or adaptive processes occurring under therapeutic pressures [170]. In DMGs, intrinsic resistance is largely driven by the extensive effects of epigenetic dysregulations on various downstream signaling pathways, tumor heterogeneity, and the immunosuppressive tumor microenvironment (TME) [171].

#### 12.4.1. Epigenetic Dysregulation

As discussed in the Section 7, H3 K27-altered mutations result in widespread epigenetic reprogramming that influences multiple downstream pathways.

These epigenetic alterations affect the expression of DNA repair mechanisms. The suppression of transcription factors such as the BReast CAncer 1 gene (BRCA 1) can significantly decrease the expression of homologous recombination repair (HRR) genes, impairing the repair of double-stranded DNA breaks [172]. In parallel, the acetylation of histone H3 plays a regulatory role in base excision repair (BER) activity. In DMGs, reduced methylation at this site leads to a corresponding increase in H3 K27 acetylation. This imbalance between acetylation and methylation ultimately results in decreased BER expression [173]. With impaired DNA repair mechanisms, H3 K27-altered DMGs can more rapidly accumulate genetic mutations and facilitate tumor progression.

Epigenetic dysregulation also promotes uncontrolled cell cycle progression. H3 K27me3 represses gene expression by compacting chromatin, while H3 K27ac relaxes chromatin structure, enabling increased gene expression. Not surprisingly, hypomethylation and acetylation have distinct downstream effects. The inhibition of H3 K27me3 effectively removes the G1/S critical cell cycle checkpoints by silencing genes such as *RB1*, *CDKN2A*, and *CDKN4/6* [91,174]. In addition, it suppresses apoptosis and promotes cellular growth through the activation of concomitant gain-of-function mutations in growth factor receptors, including *EGFR*. These growth factors can activate downstream effectors such as the PI3K/Ak strain transforming (Akt)/mTOR axis, allowing for tumor growth [175,176].

H3 K27ac leads to the upregulation of certain pathways, including *PDGFR* and *MYC* signaling, both of which drive tumor growth and expansion [177]. The upregulation of these pathways can generate a positive feedback loop because the acetylated site keeps the chromatin open, allowing for the prolonged binding of promoter and enhancer transcription factors [174]. The switch/sucrose nonfermentable (SWI/SNF) chromatin remodeling complex can further antagonize PRC2 function, simultaneously increasing H3 K27ac while reducing H3 K27me3, thereby reinforcing this epigenetic state [178].

#### 12.4.2. Tumor Heterogeneity

DMGs exhibit intratumoral heterogeneity, with distinct subclonal populations demonstrating variable therapeutic sensitivity. Although the extent of this heterogeneity is incompletely defined, given limited studies characterizing DMG-specific subclonal architecture, certain subpopulations appear intrinsically more resistant to treatment [179].

One study identified a subpopulation of aldehyde dehydrogenase-positive (ALDH+) tumor cells. These ALDH+ cells demonstrate increased transcription of MYC, DNA damage repair genes, Early-2-Factor (E2F) targets, glycolysis-related genes, and components of the mTOR pathway, enabling the enhanced repair of RT-induced damage [180]. Additional resistant subpopulations may arise within hypoxic tumor regions. Hypoxia can also induce the expression of hypoxia-inducible factors (HIFs) in a subpopulation of tumor cells. HIFs activate enzymes that support tumor survival under low-oxygen stress. Because effective RT relies on adequate oxygenation to generate oxidative damage, hypoxic tumors are less responsive to RT [181].

Other adaptive mechanisms involve intracellular communication through small extracellular vesicles (sEVs) that subpopulations of tumor cells can develop. sEVs can be released, for example, upon exposure to RT and contain proteins, microRNAs (miRNAs) and metabolites associated with glycolysis, oxidative phosphorylation, and DNA repair. The uptake of these vesicles by surrounding cells may enhance mitochondrial metabolism and contribute to treatment resistance [182].

#### 12.4.3. Immunosuppressive Tumor Microenvironment

The “cold” TME is defined by minimal immune-cell infiltration, a low expression of pro-inflammatory cytokines, and a low tumor mutational burden (TMB). Together, these features contribute to resistance to many standard therapies [183,184].

Recent single-cell and spatial transcriptomic analyses suggest that oligodendrocyte precursor cells (OPCs) are the predominant cells of origin for DMG [185,186,187,188,189]. In the presence of the H3 K27-altered mutations, these OPC-like tumor cells continue to proliferate, but further glial differentiation is stopped [190,191]. This arrested differentiation suppresses innate and adaptative immune responses through the secretion of immunosuppressive cytokines such as transforming growth factor-β (TGF-β). TGF-β promotes immunosuppressive signaling in surrounding myeloid cells and reduces the production of pro-inflammatory cytokines [192].

Glioma-associated myeloid cells (GAM) are the most abundant non-malignant cell population in the tumor microenvironment and consist primarily of microglia and monocyte-derived macrophages [179]. These GAM cells are ineffective at recruiting or activating T cells, thereby limiting antitumor immune responses [190]. As a result, the tumor environment lacks the presence of T cells. With no T cells present within the tumor, immune checkpoint inhibitors targeting PD-1, PD-L1, or cytotoxic T-lymphocyte-associated antigen-4 (CTLA-4), which are present on T cells, are ineffective in DMGs [193,194,195]. Dexamethasone also suppresses the expression of CTLA-4 in DMG, compounding resistance to immunotherapy [183].

Additionally, although the H3 K27-altered mutations lead to several downstream effects on gene transcriptions, DMG generates relatively few neoantigens for immune recognition. This low TMB further reduces immunogenicity and facilitates immune evasion [196].

### 12.5. Molecular Mechanisms of Therapeutic Resistance

DMGs harbor several molecular alterations that confer resistance to chemotherapy, targeted therapies, and RT. As discussed previously, MGMT promoter methylation is infrequent in DMGs. Unmethylated *MGMT* results in the expression of a DNA repair enzyme that removes alkyl groups from guanine bases, reversing the DNA damage caused by alkylating agents such as temozolomide. As a result, temozolomide is largely ineffective in these tumors [197]. Resistance to temozolomide may be further amplified in DMGs harboring *TP53* mutations. In glioblastoma, the mutant p53 exhibits gain-of-function activity that upregulates MGMT expression. The knockout of mutant *TP53* markedly reduces MGMT levels and increases temozolomide sensitivity fivefold, suggesting that *TP53*-mutant DMGs may be particularly resistant to alkylating chemotherapy [198,199]. In response to DNA damage, p53-deficient cells exhibit ~90% clonogenic survival compared with ~50% in p53-proficient cells. Chemotherapy resistance correlated with accelerated DNA repair in p53-deficient cells [200].

Mutations in *PDGFRA* and *EGFR* can also contribute to chemotherapy resistance. *PDGFRA* amplification drives aggressive tumor behavior through the activation of PI3K and MAPK signaling and downstream oncogenic programs involving *N-MYC*, sex determining region Y-box 2 (*SOX2*), nuclear factor kappa-light-chain-enhancer of activated B cells (NF-κB), and Signal Transducer and Activator of Transcription 3 (*STAT3*) [201,202]. Despite being treated with avapritinib, a next-generation tyrosine kinase inhibitor of *PDGFRA*, *PDGFRA*-mutated DMG can still develop resistance through metabolic reprogramming. By upregulating peroxisome proliferator-activated receptor alpha (*PPAR-α*) and sterol regulatory element binding protein-1 (SREBP1/2), tumor cells can survive treatment by switching to fatty acid metabolism and oxidative phosphorylation [203,204].

A common mutant variant of *EGFR* in gliomas, and therefore present in a subset of DMGs, is *EGFRvIII*. This variant does not require ligand binding for activation and is constitutively active, with downstream effects on several signaling pathways including PI3K, MAPK, and STAT3 pathways, and proto-oncogene tyrosine-protein kinase (Src) family kinase [205]. Targeted therapies for EGFR have been developed with minimal effects on treating gliomas because EGFR-mutated tumors are capable of undergoing molecular reprogramming. For example, EGFR inhibition can trigger rapid adaptive responses. By blocking EGFRvIII, tumor cells can adapt and activate an alternative pathway by upregulating TNF. TNF can active the c-Jun N-terminal kinase (JNK), Axl Receptor Tyrosine Kinase (Axl), and Extracellular Signal-Regulated Kinase (ERK) signaling axis that promotes primary resistance and overcomes EGFR inhibition [206]. Additionally, when EGFRvIII is blocked, tumor cells can upregulate phosphoinositide 3-kinase p110 delta isoform (PI3Kp110δ), which mediates sensitivity to EGFR inhibitors like erlotinib [207]. After anti-EGFR treatment, stressed glioma cells can secrete TGF-β which activates yes-associated protein (*YAP*) nuclear translocation and snail family transcriptional repressor 2 (*SNAI2*) expression, driving mesenchymal trans-differentiation into a state independent of EGFR signaling [208]. *EGFR* amplification and phosphatase and tensin homolog (*PTEN*) loss can also enhance DNA repair machinery and activate RAS-dependent PI3K/Akt and MAPK signaling, further contributing to resistance to both chemotherapy and RT [209,210].

## 13. Therapeutic Monitoring and Response Assessment

With the investigation of new therapies come novel ways of monitoring response to treatment. Though surveillance MRIs can show radiographic response to treatment in a non-invasive manner, at times it is difficult to gather whether there is radiographic pseudoprogression from recent RT or immunotherapies versus true progression while on treatment versus pseudoresponse (for example, if a patient is receiving bevacizumab which can contribute to radiographic pseudoresponse). Additionally, the standard response (RANO-HGG) criteria were not specifically designed for DMGs and have limitations, particularly in the era of CAR T-cell and targeted therapies that alter tumor biology without inducing immediate volumetric changes, or in the case of CAR T-cell and other immunotherapies due to the resulting inflammatory response that can mimic progression. Advanced imaging techniques such as MET PET, FET, F-DOPA, and ^18^F-fluciclovine PET are increasingly being utilized in distinguishing tumor progression from pseudoprogression, particularly when conventional MRI findings are equivocal [68]. There is an ongoing clinical study evaluating the use of ^18^F-fluciclovine PET in children with HGGs including DMGs to differentiate progression from post-treatment changes (Study ID: NCT05553041).

ctDNA in the plasma or CSF can serve not only as a diagnostic tool but also as a critical objective adjunct for longitudinal disease monitoring. A decrease in plasma ctDNA levels was demonstrated following RT, while subsequent progression on MRI correlated with an increase in ctDNA, indicating its utility as a minimally invasive tool for serial response assessment [43]. This molecular monitoring was also shown in the integrated analysis of patients receiving dordaviprone, which utilized serial CSF collection. The study showed that an increase in ctDNA preceded radiographic tumor progression in some patients, and those who displayed a decrease in ctDNA over time had longer PFS [211], indicated that monitoring ctDNA can contribute to prognostic evaluation. Currently, there are several ongoing clinical trials, including CAR T-cell studies and combination trials of dordaviprone, evaluating liquid biopsy biomarkers to measure treatment response and outcome [212].

## 14. Conclusions

Significant advances in characterizing the molecular and epigenetic architecture of H3 K27-altered DMGs have been achieved, but translating these insights into durable clinical survival remains a challenge. The August 2025 FDA approval of dordaviprone (ONC201) for progressive DMGs harboring an H3 K27M mutation represents a notable milestone; however, its clinical impact is tempered by an ORR of approximately 20%, highlighting the fact that a significant number of patients still lack effective therapeutic options. As reflected by the diverse clinical trials summarized in Table 2, the field is actively exploring cellular immunotherapies, vaccines, combination targeted and radiation therapies, and novel locoregional delivery approaches to overcome key barriers outlined in this review. Additionally, improving response assessments, including the incorporation of advanced neuroimaging and ctDNA, is crucial in identifying responders and refining future trial designs. Ultimately, while the approval of dordaviprone provides a long-awaited systemic benchmark, substantial progress in the management of H3 K27-altered DMGs will depend on effectively translating biologic insights into durable therapeutic benefit through coordinated, international, multi-institutional efforts.

## Figures and Tables

**Table 1:** Summary of available and investigational therapies for DMG.

Treatment Category	Therapeutic Agent	Mechanism of Action	Clinical Setting	Key Efficacy Signal	Limitations
Conventional Therapies	Radiation therapy	Generation of DNA strand breaks and ROS inducing cell death	Standard of care, used as first-line treatment	Median PFS: 7–10 months Median OS in adults: 9–13 months Median OS in children: 8–11 months	Acute side effects: fatigue, headache, alopecia Early delayed side effects: pseudoprogression Late-delayed side effects: neurocognitive and endocrine dysfunction Eventual radioresistance and near universal tumor recurrence
	Temozolomide Lomustine	Alkylating DNA, causing cross-linking, double strand breaks, and cell death	Used in the adjuvant setting post-RT or for recurrent tumors	Larger systematic reviews showed no survival benefit from chemotherapy	MGMT promoter unmethylation limits efficacy Lack of durable response
Imipridones	Dordaviprone (ONC201)	Simultaneous selective antagonist of DRD2 and allosteric agonist of mitochondrial protease ClpP, leading to an ISR and cell death	FDA-approved for recurrent H3 K27M-altered DMG in adults	Median OS 13.7 months and median DOR 11.2 months in adults ORR of 20%	Limited pediatric efficacy data
	ONC206	Greater potency in preclinical studies relative to dordaviprone, leading to an ISR and cell death	Recurrent H3 K27-altered DMG and being investigated in early Phase 1 clinical trials	Survival benefit in patient-derived models of DMG	No clinical efficacy data available
	ONC212	GPR132 receptor agonist and increased potency relative to dordaviprone; GPR132 functions as tumor suppressor	Only used in preclinical studies	Induction of apoptosis in DMG cells, especially with combined therapy	No clinical data
Targeted Therapies	Panobinostat	Histone deacetylase inhibitor that increases global histone acetylation, counteracting epigenetic dysregulation caused by the H3 K27M mutation	Clinical trials for newly diagnosed and recurrent H3 K27-altered DMG	Stable disease as best response in small single-center studies; improved outcomes with local delivery in Phase 1 trials	Systemic toxicity limits dosing Paradoxical adaptive responses Delivery challenges
	Ribociclib, Abemaciclib	CDK4/6 inhibitor prevents phosphorylation of Rb protein, ultimately causing cell cycle arrest	Clinical trials for newly diagnosed and recurrent H3 K27-altered DMG	Cytostatic effects in patient-derived models of DMG	Limited clinical data Limited objective response
	Infrigatinib, Pemigatinib	FGFR inhibition targeting MAPK/mTOR pathway activation	Clinical trials in FGFR-altered gliomas	Durable disease stabilization in CNS tumors harboring FGFR point mutations	Limited BBB penetration Modest efficacy
	Tazemetostat	EZH2 inhibitor that prevents catalysis of mono-, di-, and trimethylation of H3 K27 and induces tumor suppressor protein p16Ink4a	Only used in preclinical studies	Tumor cell growth abolished in mouse and patient-derived DMG models	No clinical data in H3 K27-altered tumors Limited BBB penetration EZH2 may have paradoxical tumor suppressor function
	PDGFRA inhibitors (e.g., avapritnib)	Inhibition of PDGFRA signaling, targeting downstream PI3K/Akt and MAPK pathways	Clinical trials in PDGFRA-altered gliomas, including H3 K27-altered DMG	Preclinical activity and target engagement in molecularly selected tumors	Limited clinical efficacy Adaptive resistance CNS penetration challenges
	Selinexor	XPO1 inhibitor that binds to key exporter of molecules from the nucleus to cytoplasm, inducing cell cycle arrest and death	Being investigated in clinical trials for adult and pediatric HGGs	Disease stabilization in pediatric patients with high-grade gliomas, including H3 K27M-mutant	No objective response No prospective clinical data specifically for H3 K27-altered tumors
Anti-Angiogenic Therapy	Bevacizumab	Inhibits tumor angiogenesis	Being investigated in clinical trials for newly diagnosed and recurrent or progressive CNS tumors in children and adults	HERBY trial (pediatric HGG): no improvement in EFS with Bevacizumab + RT + TMZ vs. RT + TMZ alone Temporary symptom palliation	Limited objective response No survival benefit
Immunotherapy	GD2 CAR T-cell therapy	Targets GD2 disialoganglioside expressed on DMG cells, triggering cytokine generation and inducing tumor cell death	Being investigated in early-phase clinical trials	Measurable tumor volume reduction; symptom improvement	Neurotoxicity Limited persistence of CAR T-cells due to immunosuppressive TME
	H3K27M peptide vaccine	Mutation-specific long peptide vaccine induces immune system response against H3K27M neoantigen	Being investigated in clinical trials for children and adults with H3 K27-altered DMG	Durable response in 1 of 8 adult patients in first-in-human study	Immunosuppressive TME Detrimental effect of corticosteroids on immune responses
	Nivolumab, Pembrolizumab	Block the interaction between PD-1 expressed on T-cells and PD-L1 expressed on tumor cells, allowing for antitumor immune response	Clinical trials for newly diagnosed and recurrent CNS tumors in children and adults	No survival benefit in retrospective studies Efficacy mainly in hypermutant tumors	Immunosuppressive TME No prospective clinical data specifically for H3 K27-altered tumors
Drug Delivery Approaches	CED	Direct intratumoral delivery bypassing the BBB	Clinical trials and preclinical studies	Improved local drug concentration and target engagement	Invasive Technically complex Variability in distribution

Abbreviations: DMG = diffuse midline glioma; CNS = central nervous system; OS = overall survival; DNA = deoxyribonucleic acid; RT = radiation therapy; *MGMT* = methylguanine-DNA methyltransferase; DRD2 = dopamine receptor D2; ClpP = caseinolytic protease P; ROS = reactive oxygen species; ISR = integrated stress response; FDA = Food and Drug Administration; DOR = duration of response; ORR = objective response rate; GPR132 = orphan G coupled protein receptor; Rb = retinoblastoma; CED = convection-enhanced delivery; CDK = cyclin dependent kinase; *FGFR* = fibroblast growth factor receptor; EZH2 = enhancer of zeste homolog 2; XPO1 = exportin-1; *PDGFRA* = platelet-derived growth factor receptor alpha; PI3K = phosphoinositide 3-kinase; Akt = protein kinase B; MAPK = mitogen-activated protein kinase; HGG = high-grade glioma; EFS = event-free survival; CAR T-cell = chimeric antigen receptor T-cell; GD2 = disialoganglioside 2; TME = tumor microenvironment; BBB = blood–brain barrier; mTOR = mechanistic target of rapamycin; PD-1 = programmed cell death-1; PD-L1 = programmed cell death ligand 1.

**Table 2:** Active clinical trials accruing patients with DMG or DIPG.

NCT Identification Number	Title	Phase	Study Type	Description
NCT05580562	ONC201 in H3 K27M-mutant Diffuse Glioma Following Radiotherapy (the ACTION Study)	3	RCT Multicenter, including international sites	Randomized, double-blind, placebo controlled, parallel-group, international study in patients with newly diagnosed H3 K27M-mutant diffuse glioma to assess whether treatment with ONC201 radiotherapy will extend OS and PFS
NCT05009992	Combination Therapy Trial Using an Adaptive Platform Design for Children and Young Adults with DMGs including DIPGs at Initial Diagnosis, Post-Radiation Therapy and at Time of Progression (PNOC022)	2	Open-label Multi-arm Multicenter, including international sites	Efficacy of the combination of ONC201 with a novel agent for treating patients with DMG in an adaptive design
NCT06894979	Testing the Addition of an Anti-Cancer Drug, AZD1390, During Radiation Therapy for Newly Diagnosed High-Grade Glioma, Diffuse Midline Glioma, or Diffuse Intrinsic Pontine Glioma	1	Dose-escalation	Study of the side effects and best dose of AZD1390 when given together with radiation therapy for the treatment of pediatric patients with high-grade glioma, DMG, or DIPG
NCT04510051	Chemotherapy and CAR-T Cell Immunotherapy for the Treatment of IL13Ralpha2 Positive Recurrent or Refractory Brain Tumors or Newly Diagnosed DIPG/DMG	1	Dose-escalation	Study the side effects of CAR-T cell immunotherapy in combination with chemotherapy in patients with newly diagnosed DIPG or DMG
NCT05843253	Ribociclib and Everolimus Following Radiotherapy in Patients with High-Grade Glioma, and Diffuse Intrinsic Pontine Glioma, Harboring Cell Cycle and/or PI3K/mTOR Pathway Genetic Changes (TarGet Trial)	2	Open-label Multi-arm Multicenter	Efficacy of ribociclib and everolimus after radiation therapy in treating pediatric and young adult patients with high-grade glioma or DIPG that have mutations in cell cycle/PI3K/mTOR pathways
NCT04732065	ONC206 for the Treatment of Newly Diagnosed or Recurrent Diffuse Midline Gliomas or Other Recurrent Primary Malignant Central Nervous System Tumors	1	Dose-escalation	Study of the effects and best dose of ONC206 alone or in combination with radiation therapy in patients with newly diagnosed or recurrent DMG, or other recurrent primary malignant central nervous system tumors
NCT04541082	Phase I Study of Oral ONC206 in Recurrent and Rare Primary Central Nervous System Neoplasms	1	Dose-escalation	Evaluation of safety and tolerability profile and evaluation of the occurrence of dose-limiting toxicities (DLTs) following single weekly or multiple-day weekly dose regimens of oral ONC206 in patients with recurrent primary central nervous system (CNS) neoplasms
NCT05099003	A Study of the Drug Selinexor with Radiation Therapy in Patients With Newly Diagnosed DIPG and High- Grade Glioma (HGG)	1/2	Dose-escalation	Evaluation of the safety, side effects, and best dose of selinexor given in combination with standard radiation therapy in treating children and young adults with newly diagnosed DIPG or high-grade glioma (HGG) with H3 K27M mutation
NCT05096481	A Peptide Vaccine (PEP-CMV) for the Treatment of Pediatric Patients with Newly Diagnosed High-Grade Glioma and Diffuse Intrinsic Pontine Glioma, or Recurrent, Refractory, or Progressive Medulloblastoma	2	Non-randomized Open-label	Evaluation of the efficacy of a peptide vaccine (PEP-CMV) in treating pediatric patients with newly diagnosed high-grade glioma, DIPG, or medulloblastoma that is recurrent, refractory to treatment, or progressive
NCT04943848	A Vaccine (Rhsc-Dipgvax) in Combination with Immunotherapy (Balstilimab and Zalifrelimab) for Treatment of Diffuse Intrinsic Pontine Glioma and Diffuse Midline Glioma in Pediatric Patients	1	Dose-escalation	Study of the safety and side effects of the combination of rHSC-DIPGVax, balstilimab and zalifrelimab, and determination of the best dose of zalifrelimab in this combination for treating children with DIPG and DMG
NCT02644460	Abemaciclib and Radiation Therapy in Treating Younger Patients with Newly Diagnosed Diffuse Intrinsic Pontine Glioma, Recurrent or Refractory Solid Tumors, or Malignant Brain Tumors	1	Dose-escalation	Study of the side effects and best dose of abemaciclib when given with radiation therapy in treating younger patients with newly diagnosed DIPG, recurrent/refractory solid tumors, or malignant brain tumors
NCT05413304	Abemaciclib Neuropharmacokinetics of Diffuse Midline Glioma Using Intratumoral Microdialysis	1	Non-randomized Open-label	Evaluation of the safety and feasibility of intratumoral microdialysis placement post-high-grade glioma resection or midline glioma biopsy
NCT05077735	Stereotactic Biopsy Split-Course Radiation Therapy in Diffuse Midline Glioma, SPORT-DMG Study	2	Interventional Single-arm	Study of the clinical outcomes of hypofractionated radiation therapy in patients with DMG
NCT06838676	ACT001 for the treatment of Newly Diagnosed Diffuse Intrinsic Pontine Gliomas (DIPG) and Progressive, Refractory, or Recurrent DIPG or H3K27-Altered High-Grade Gliomas	1/2	Interventional Multicenter	Efficacy of ACT001 in patients with newly diagnosed DIPG, and DIPG and H3K27-altered high-grade gliomas that are progressive, recurrent, or refractory
NCT04196413	GD2CART for the Treatment of H3 K27M Mutated Diffuse Intrinsic Pontine Glioma or Spinal Diffuse Midline Glioma	1	Dose-escalation	Study of the side effects and best dose of GD2 CAR T cells and their efficacy in treating patients with DMG or spinal diffuse midline glioma
NCT04099797	Genetically Engineered Cells (C7RGD2.CAR T Cells) for the Treatment of Patients with GD2-Expressing High Grade Glioma or Diffuse Intrinsic Pontine Glioma (GAIL-B Trial)	1	Open-label Non-randomized	Study of the side effects and best dose of C7R-GD2.CAR T cells in treating patients with GD2-expressing high-grade glioma or DIPG
NCT05478837	Genetically Modified Cells (KIND T Cells) for the Treatment of HLAA*0201-Positive Patients with H3.3 K27M-Mutated Diffuse Midline Glioma	1	Dose-escalation	Study of the safety, side effects, and best dose of genetically modified cells called KIND T cells after lymphodepletion (a short dose of chemotherapy) in treating patients who are HLA-A*0201-positive and have H3.3 K27M-mutated diffuse midline glioma
NCT04185038	Genetically Engineered Cells (B7-H3- Specific CAR T Cells) in Treating Pediatric Patients with Diffuse Intrinsic Pontine Glioma, Diffuse Midline Glioma, or Recurrent or Refractory Central Nervous System Tumors	1	Open-label Non-randomized	Study of the side effects, best dose, and efficacy of genetically engineered cells CAR T cells in treating patients with DIPG, DMG, or recurrent/refractory central nervous system tumors
NCT05762419	Non-Invasive Focused Ultrasound with Oral Etoposide for the Treatment of Children with Progressive Diffuse Midline Glioma	1	Open-label Non-randomized	Evaluation of the feasibility of safely opening the blood–brain barrier in children with progressive DMG treated with oral etoposide using focused ultrasound with microbubbles and neuro-navigator-controlled sonication
NCT07076498	Engineered HSV-1 M032 for the Treatment of Children and Adults with Newly Diagnosed Diffuse Midline Glioma After Standard of Care Radiation	1	Dose-escalation Dose-expansion	Assessment of the safety and tolerability of IL-12expressing HSV-1 NSC 733972 (M032) administered intratumorally via stereotactic intratumoral injection in children and adults with DMG after receiving standard-of-care radiation
NCT06639607	PEP-CMV + Nivolumab for Newly Diagnosed and Recurrent DMG/High-grade Glioma, Medulloblastoma, and Ependymoma (PRiME II)	1/2	Multicenter Interventional	Study of the safety, immunogenicity, and efficacy of a CMV-directed peptide vaccine plus checkpoint blockade
NCT06305910	CD200AR-L and Allogeneic Tumor Lysate Vaccine Immunotherapy for Recurrent HGG and Newly Diagnosed DMG/DIPG in Children and Young Adults	1	Dose-escalation	Determination of the maximum tolerated dose of CD200AR-L, a new adjuvant CD200 activation receptor ligand, when given with a fixed dose of GBM6-AD vaccine, imiquimod, and a single dose of radiation for patients with recurrent high-grade glioma (HGG) or following standard-of-care therapy radiation therapy for newly diagnosed DIPG/DMG
NCT06624371	Atovaquone Combined with Radiation in Children with Malignant Brain Tumors (AflacBT2303)	1	Open-label Non-randomized	Study of the safety and tolerability of atovaquone in combination with standard radiation therapy (RT) for the treatment of pediatric patients with newly diagnosed pediatric high-grade glioma/DMG/DIPG
NCT05768880	Study of B7-H3, EGFR806, HER2, And IL13-Zetakine (Quad) CAR T Cell Locoregional Immunotherapy for Pediatric DIPG, DMG, and Recurrent Or Refractory Central Nervous System Tumors	1	Open-label Non-randomized	Study of CNS locoregional adoptive therapy with SC-CAR4BRAIN delivered via an indwelling catheter into the ventricular system in children and young adults with DIPG, DMG, and recurrent or refractory CNS tumors.
NCT07223034	A Study of 177Lu-PSMA-617 in People with Gliomas (LU-TARGET)	1	Open-label Non-randomized	Investigation of whether the radiopharmaceutical therapy (RPT) 177LuPSMA-617 is a safe treatment for people with IDH wildtype glioma
NCT06357377	A Study of the Safety, Dosing, and Delivery of NEO100 in Patients with Pediatric Brain Tumors	1	Dose-escalation	Study of the safety of intranasal NEO100 in patients with pediatric-type diffuse high-grade gliomas

Abbreviations: RCT—Randomized Controlled Trial; OS—Overall Survival; PFS—Progression-Free Survival; DMG—Diffuse Midline Glioma; DIPG—Diffuse Intrinsic Pontine Glioma; HSV—Herpes Simplex Virus; IL—Interleukin; CMV—Cytomegalovirus; PSMA—Prostate-Specific Membrane Antigen; PI3K—Phosphoinositide 3 Kinase; mTOR—Mechanistic Target of Rapamycin; HLA—Human Leukocyte Antigen.

## Data Availability

No new data were created or analyzed in this study.

## Abbreviations

Lys—Lysine; Met—Methionine; *EZHIP*—Enhancer of Zeste Homologs Inhibitory Protein; *EGFR*—Epidermal Growth Factor; *TP53*—Tumor Protein 53; *ACVR1*—Activin A Receptor Type 1; *PDGFRA*—Platelet-Derived Growth Factor Receptor Alpha; *NF1*—Neurofibromin 1; *ATRX*—Alpha Thalassemia/Mental Retardation; *PPM1D*—Protein Phosphatase Mg^2+^/Mn^2+^-Dependent1D; *CDK4/6*—Cyclin-Dependent Kinase 4 and 6; *PIK3CA*—Phosphatidylinositol-4,5-Bisphosphate 3-Kinase Catalytic Subunit Alpha; *PIK3R1*—Phosphatidylinositol 3-Kinase Regulatory Subunit 1.
